# Management of End-stage Renal Disease Associated with Systemic Rheumatic Diseases

**DOI:** 10.31662/jmaj.2019-0020

**Published:** 2019-09-10

**Authors:** Suguru Honda, Yasuhiro Katsumata, Kazunori Karasawa, Hisashi Yamanaka, Masayoshi Harigai

**Affiliations:** 1 Department of Rheumatology, School of Medicine, Tokyo Women’s Medical University, Tokyo, Japan; 2 Department of Nephrology, School of Medicine, Tokyo Women’s Medical University, Tokyo, Japan; 3 Division of Epidemiology and Pharmacoepidemiology, Department of Rheumatology, School of Medicine, Tokyo Women’s Medical University, Tokyo, Japan

**Keywords:** rheumatic disease, hemodialysis, end-stage renal disease, immunosuppressant agents, team approach

## Abstract

The outcomes of rheumatic diseases (RDs) have improved over the past decades. However, a significant proportion of the patients still suffer from end-stage renal disease (ESRD) and have to bear the burden of hemodialysis. It is crucial to prevent patients with RDs from developing ESRD from viewpoints of medicine and medical economics. For those who already have ESRD, it is important to improve vial prognosis and quality of life through appropriate management of disease activity and comorbidities related to ESRD. Thus, rheumatologists and nephrologists need to recognize risk factors associated with progression to ESRD along with their appropriate management. Although the activity of most RDs tends to decrease after initiation of hemodialysis, disease activity may still increase, and recognizing how to appropriately use immunosuppressive agents even after the development of ESRD is crucial. The treatment of RDs needs extra attention as hydroxychloroquine requires more frequent monitoring for adverse drug reactions; therapeutic drug monitoring is necessary for mycophenolate mofetil, cyclosporine A, and tacrolimus; cyclophosphamide and azathioprine need dose adjustments; methotrexate and bucillamine are contraindicated in patients with ESRD; leflunomide and sulfasalazine do not require significant dose reduction and iguratimod should be carefully administered. The pharmacokinetics of biological agents such as rituximab or belimumab are not affected by ESRD, and dose adjustments are not necessary. Collaboration between rheumatologists and nephrologists is needed more than ever and is expected to produce a complementary effect and achieve better outcomes in clinical settings, although this cooperation has not always been conducted appropriately.

## Introduction

The kidney is a vital organ that can be damaged by a variety of rheumatic diseases (RDs). Despite progress in the diagnosis and treatment of RDs, with improved survival rates over the past few decades ^(1)^, many patients lose renal function and some progress to end-stage renal disease (ESRD). Major systemic RDs that may lead to ESRD and their representative examples are summarized in Table 1. As of 2014, patients with lupus nephritis (LN) and rapidly progressive glomerulonephritis accounted for 1.7% of Japanese patients on maintenance dialysis ^(2)^.

**Table 1.** Major Systemic Rheumatic Diseases Leading to ESRD and Their Representative Examples.

Diseases	Representative examples
SLE	Lupus nephritis
Systemic sclerosis	Scleroderma renal crisis
AAV	ANCA-associated glomerulonephritis, RPGN
anti-GBM disease	anti-GBM glomerulonephritis, RPGN
IgA vasculitis	IgA vasculitis nephritis
Amyloidosis	Amyloid nephropathy
Drug-induced	Calcineurin inhibitor nephrotoxicity

SLE = Systemic lupus erythematosus, AAV = ANCA-associated vasculitis, RPGN = rapidly progressive glomerulonephritis, anti-GBM Ab = anti-glomerular basement membrane antibody, ESRD = end-stage renal disease

Development of ESRD is associated with a high mortality rate and poses clinical challenges to rheumatologists and nephrologists. In patients with ESRD, these specialists face difficulties in evaluating the activity of RDs, treating disease relapses due to the restrictions in using certain medications, and managing comorbidity, including infections and cardiovascular diseases. Thus, rheumatologists and nephrologists need to re-recognize the challenges in managing patients with ESRD associated with RDs and the importance of cooperation with each other. In clinical settings, however, this cooperation has not always been conducted appropriately. Considering the gap between the goals and the current status, we described the epidemiology of ESRD in patients with RDs in the first part, treatment with renocardiovascular protective agents, immunosuppressive agents, and immunomodulators in the second part, and collaboration of rheumatologists and nephrologists in the management of ESRD patients in the last part of this review.

## Epidemiology of ESRD in Patients with RDs

### SLE

#### Incidence of ESRD

Analysis of the Italian multicentric retrospective cohort of 499 patients from 1970 to 2016 showed that renal survival in the recent 15 years progressively improved ^(3)^. Another meta-analysis of 18,309 patients showed that the risk of ESRD decrease until the mid-1990s and then plateaued only in developed countries, which may reflect limited access to and effectiveness of treatment ^(4)^.

#### Risk factors for ESRD

Male sex may be associated with a poor prognosis for clinical outcomes of LN and progression to ESRD ^(5)^. Faurschou et al. reported that nephritis symptoms for more than 6 months prior to biopsy, serum creatinine of >140 μmol/L or 1.83 mg/dL, diffuse proliferative glomerulonephritis, and tubular atrophy were associated with progression to ESRD by multivariate regression analyses in an unselected cohort of patients with LN ^(5)^. Korbet et al. reported that in patients with severe LN, the presence of anti-Ro antibody and failure to achieve remission were predictive factors for ESRD ^(6)^. The utility of histopathological characteristics in predicting prognosis is controversial. Rijnink et al. ^(7)^ reported that ESRD was associated with fibrinoid necrosis (hazard ratio [95% confidence interval, CI], 1.08 [1.02 to 1.13]), fibrous crescents (hazard ratio [95% CI], 1.09 [1.02 to 1.17]), and interstitial fibrosis/tubular atrophy of ≥25% (hazard ratio [95% CI], 3.89 [1.25 to 12.14]). Another systematic review showed that the 15-year risk of ESRD in patients with class IV LN was 44% in developed countries ^(4)^. On the other hand, International Society of Nephrology/Renal Pathology Society class was not significantly associated with overall renal survival. Lack of access to medical care can limit preventive treatment. A population-based ecological design study by ZIP code in California during 1999–2004 reported that patients without insurance, with Medicaid, or with high rates of avoidable hospitalizations showed a relatively high incidence of LN-related ESRD ^(8)^.

#### Disease activity in patients with dialysis

The clinical activity of SLE is quiescent in most patients with ESRD caused by LN. Mattos et al. ^(9)^ reviewed 24 retrospective studies published between 1973 and 2011 and found that 15 studies reported a substantial decrease in clinical and/or serological activity of SLE after the development of ESRD, whereas nine others showed that progression to ESRD was not associated with decreased disease activity. Cheigh et al. ^(10)^ reported that lupus activity was clinically apparent in 55.4% of patients with ESRD due to SLE during the first year, in 6.5% during the fifth year, and in 0% during the tenth year. During this period, the percentage of patients with two or more abnormal serological markers, including antinuclear antibody, anti-dsDNA antibody, 50% haemolytic complement activity, and complement 3, fell from 80% to 22%. In other studies, the most frequent recurrent manifestations were arthritis, fever, pericarditis, and pleuritis ^(11)^. These signs and symptoms also develop in non-SLE patients with hemodialysis or heart failure, and it is sometimes difficult to distinguish non-lupus symptoms from a flare of SLE. It is controversial whether serological markers, such as serum complement levels and titers of anti-dsDNA antibody, can be surrogate markers of disease activity in lupus patients with ESRD. A retrospective analysis of a long-term cohort of 32 SLE patients with ESRD showed that serological markers and hematological British Isles Lupus Assessment Group activity were the major indicators for disease activity after development of ESRD ^(12)^. Okano et al. studied SLE disease activity in 14 patients with ESRD and found that all five patients with flares had accompanying decreased serum complement levels ^(11)^. Moreover, lack of validated measures of SLE disease activity in ESRD patients makes investigation more challenging. Collectively, decision-making for treatment of ESRD patients with LN should be based on close monitoring and changes over time of onset and severity of extra-renal manifestations and values of serological markers.

### SSc

#### Incidence of ESRD

Scleroderma renal crisis (SRC) is characterized by rapidly progressive renal injury and hypertension as a result of proliferative and obliterative vasculopathy without inflammatory changes or glomerular immune deposits ^(13)^. A high proportion of patients with SRC temporarily or permanently require dialysis, although SRC itself is not common. SRC develops in about 10% of patients with diffuse scleroderma and 2% of patients with limited disease in the U.S. ^(13)^. A prospective observational cohort study enrolling 145 patients with SRC treated with angiotensin-converting enzyme (ACE) inhibitors reported that 28 patients (19%) died early within 6 months, 32 (22%) received permanent dialysis, and 24 (16%) received temporary dialysis ^(14)^. About 1 in 10 patients with SRC develops without hypertension, known as a normotensive renal crisis, that leads to worse renal outcomes and higher mortality ^(15), (16)^.

#### Risk factors for ESRD

Steen et al. ^(17)^ identified several predictive factors for SRC as follows: SSc disease duration of <4 years, diffuse cutaneous SSc, rapidly progressive skin thickening, new anemia, new cardiac events, anti-RNA-polymerase III antibody, prednisone use of >15 mg/day within the previous 3 months, and CSA therapy within the previous 3 months. On the other hand, baseline blood pressure, serum creatinine level, and proteinuria or hematuria were not risk factors for SRC ^(18)^.

Teixeira et al. reported that age of >53 years and normal blood pressure were significant risk factors for decreased dialysis-free survival in patients with SRC after multivariate analysis ^(19)^. Somewhat surprisingly, prophylactic use of ACE-inhibitors was associated with worse renal outcomes and increased likelihood of requiring permanent dialysis ^(13)^, although ACE-inhibitors such as captopril and enalapril are considered as essential medications for SRC because of improvement of survival rate ^(20), (21)^. Recurrence of SRC has been reported, but is extremely rare; less than 5% of patients with SRC who underwent renal transplantation had a recurrence ^(22)^.

#### Disease activity in patients with dialysis

One report has showed that modified Rodnan total skin thickness scores improved in all four patients after kidney transplantation with an average decline of 60.7% (p = 0.024) ^(23)^.

### Microscopic polyangiitis and granulomatosis with polyangiitis

#### Incidence of ESRD

Several studies have shown that renal disease of microscopic polyangiitis/granulomatosis with polyangiitis (MPA/GPA) progresses to ESRD within 3 to 7 years of diagnosis in 20%–30% of the patients ^(24), (25), (26)^. A large cohort of six European Vasculitis Study Group trials in patients with antineutrophil cytoplasmic antibody (ANCA)-associated vasculitis (AAV) showed an incidence of ESRD reaching 29% during the average follow-up of 7.1 years ^(24)^.

#### Risk factors for ESRD

Treatment resistance is a risk factor for progression to ESRD. A cohort study showed that female and black patients were at higher risk of treatment resistance ^(27)^. A population-based study showed that MPO-ANCA-positive patients were more likely to develop ESRD than PR3-ANCA-positive patients after adjusting for sex, age, and serum creatinine level at diagnosis ^(25)^. There was no significant difference in mortality rates between the MPO-ANCA and PR3-ANCA patients. Compared to the non-ESRD AAV patients, ESRD AAV patients received immunosuppressants less frequently, corticosteroid alone more frequently, and had shorter durations of treatment with CYC, which reflects the perception that the risk of immunosuppressive therapy outweighs potential benefits in patients with advanced renal failure without extra-renal vasculitis ^(26)^. A recent cohort analysis suggests, however, that aggressive immunosuppressive therapy should be considered in all patients with AAV because 57% of patients with a GFR of ≤10 m/min achieve renal remission ^(27)^. A retrospective study of biopsy-proven MPO-ANCA-associated glomerulonephritis showed that the 5-year renal survival in Chinese patients classified by the same four categories were 100%, 67.4%, 58.9%, and 20.7%, respectively ^(28)^. Brix SR et al. ^(29)^ proposed a risk score to predict ESRD at 36 months based on the following three parameters: percentage of normal glomeruli (more than 25% = 0 points, 10% to 25% = 4 points, less than 10% = 6 points), percentage of tubular atrophy and interstitial fibrosis (no more than 25% = 0 points, more than 25% = 2 points), and estimated glomerular filtration rate at the time of biopsy (more than 15 mL/min/1.73 m^2^ = 0 points, no more than 15 mL/min/1.73 m^2^ = 3 points). In an independent validation cohort, the risk for developing ESRD in patients with low (0 points), intermediate (2 to 7 points), and high risk (8 to 11 points) was 0%, 27%, and 78%, respectively.

#### Disease activity in patients with dialysis

The disease activity decreases after the loss of renal function in patients with AAV. In a retrospective study enrolling 452 patients with AAV, the relapse rate in patients with ESRD was 0.08 episodes per patient-year, which was significantly lower than that in patients without ESRD at 0.2 episodes per patient-year ^(26)^. A retrospective study enrolling 89 dialysis-dependent patients with MPO-ANCA-associated vasculitis reported that the incidence of relapse was low ^(30)^. Multivariate analysis demonstrated that pulmonary involvement was the strongest predictor of relapse and patient mortality (hazard ratio [95% CI], 21.4 [2.56 to 179.1], and 4.60 [2.08 to 10.2], respectively). Among deceased patients, the major causes of death were infections and cardiovascular diseases (56.8% and 29.7%, respectively). The author concluded that prolonged maintenance of immunosuppressive therapy after the development of ESRD might lead to infection and death, and should be carefully considered in light of extra-renal disease activity, including pulmonary involvement.

### IgA vasculitis/HScP

#### Incidence of ESRD

The reported incidence of renal disease ranges from 30%–70% ^(31), (32)^, usually manifesting as hematuria with or without proteinuria. A retrospective study of 250 adults patients with HSP showed that 11% of patients with HSP had developed ESRD during the 14.8 years follow-up period and only one-fifth of the patients reached clinical remission ^(31)^. Another retrospective study with a mean follow-up period of 3.9 years showed that the incidence of a 50% increase in serum creatinine is significantly higher in patients of > 65 years old compared to patients of < 65 years old ^(33)^.

#### Risk factors for ESRD

The presence of nephrotic/nephritic syndrome and renal impairment has been shown to be associated with a poor prognosis of HScP. A long-term observational study of 114 patients with HSP compared clinical characteristics at onset between those with unfavorable and favorable outcomes ^(34)^. The unfavorable group, which included 15 patients with active renal disease and five with renal failure, had more clinical features such as nephrotic syndrome, decreased factor XIII activity, hypertension, and renal failure than the favorable group, which included 69 patients with normal urine and 25 with minor urinary abnormalities. Another retrospective study reported that age older than 7 years, severe abdominal pain, and persisting purpura were also risk factors for the presence of gross or microscopic hematuria by multivariate analysis ^(35)^, which had been demonstrated as one of the major risk factors of ESRD in children with HScP. A retrospective study investigating the differences between children (≤20 years) and adults (>20 years) with HScP showed that adults had more frequent and more severe renal involvement, less frequent abdominal pain and fever, and more frequent joint symptoms ^(36)^. The renal pathological classification of the International Study for Kidney Diseases in Children assesses prognosis according to extracapillary proliferation and crescent formation, but the classification does not consider tubulointerstitial fibrosis, endocapillary hypercellularity, arteriolar damage, or segmental sclerosis, all of which may also predict chronic kidney disease (CKD) ^(37)^.

#### Disease activity in patients on dialysis

Although recurrence of glomerular IgA deposits after kidney transplantation was documented in 7 of the 21 grafts (33%) in patients with HScP nephritis, the rate of graft loss was 2.5% during the first 5 years of follow-up ^(38)^. Data on the recurrence of HScP after initiation of dialysis are scarce.

### Anti-GBM antibody disease

#### Incidence of ESRD

The incidence of anti-GBM disease accounts for 1%–5% of all types of glomerulonephritis in an old report ^(39)^. On the other hand, Japan Renal Biopsy Registry enrolling 7,442 patients who underwent renal biopsy between 2009 and 2010 showed that only 24 patients (0.3%) had anti-GBM-antibody-positive nephritis ^(40)^. The overall survival rate was 72.7% at 1 year, and the renal survival rates at 2 months and 1 year were 35.8% and 25.0%, respectively ^(41)^. These poor overall and renal survival rates are a result of severe disease activity at presentation and rapid progression.

#### Risk factors for ESRD

Anti-GBM disease occurs more commonly in white people than in black people. The age distribution is bimodal with peaks at 20–30 and 60–70 years ^(42)^. The genetic change most closely linked to anti-GBM disease is HLA-DRB1*15:01 ^(43)^. Cui Z et al. ^(44)^ reported that serum concentrations of anti-GBM antibody and double positivity for ANCA and anti-GBM antibodies are associated with death. Several studies showed that oligoanuria at diagnosis was the best predictive factor of mortality, although pulmonary hemorrhage and dialysis dependence were not associated with mortality ^(45), (46)^. A retrospective review in patients with anti-GBM disease who received plasma exchange, prednisolone and cyclophosphamide showed that dialysis-dependent renal failure and creatinine concentration of >500 μmol/L or 5.7 mg/dL were associated with low renal survival rate ^(47)^.

#### Disease activity in patients with dialysis

Anti-GBM antibody disease is generally monophasic, and most patients who recover from the acute illness will be cured. Recurrence of clinical features after disappearance of the autoantibody is extremely rare, although relapse of clinical features while autoantibodies are still present is common ^(48)^.

## Treatment with Renocardiovascular Protective Agents, Immunosuppressive Agents, and Immunomodulators

The benefit of the renin-angiotensin-aldosterone system (RAAS) blockade for blood pressure control in patients with CKD and hemodialysis is controversial because most trials evaluating the renocardiovascular benefit of RAAS blockade exclude patients with ESRD ^(49), (50), (51)^. In a review article, Slomka et al. ^(51)^ recommend that RAAS blockade should be taken into consideration in patients with ESRD diagnosed as having heart failure and left ventricular hypertrophy, while paying attention to hyperpotassemia.

The benefits of statins in patients with CKD is also controversial ^(52)^. However, statins should be considered in patients who have multiple risk factors for cardiovascular disease, because the incidence of cardiovascular disease in patients with RDs is higher than in the control population ^(52)^. It is important to note that most immunosuppressive agents, except for azathioprine and CYC, show no intradialytic clearance with hemodialysis ^(53)^. Although safety data are limited, several immunosuppressive agents appear to be useful for reducing corticosteroid use and improving extra-renal manifestations of RDs in ESRD.

### Hydroxychloroquine

Hydroxychloroquine has been used as the standard drug for patients with SLE because of its favorable safety profile and efficacy. Hydroxychloroquine may decrease the risk of SLE flares and damage to the renal and cardiovascular systems in patients with renal involvement ^(54)^. Patients with renal disease may have unpredictably high blood drug levels because hydroxychloroquine is eliminated to a large degree through the kidney. Hydroxychloroquine-associated retinal toxicity may be increased in ESRD patients, and both dosage and monitoring frequency need to be adjusted ^(55)^.

### MMF

MMF is an inhibitor of inosine monophosphate dehydrogenase, a key enzyme of the *de novo* pathway of purine synthesis. The ester prodrug MMF is rapidly converted *in vivo* to the active drug mycophenolic acid (MPA). MMF has emerged as an alternative agent for both induction and maintenance therapy in LN ^(54)^. Metabolism of MMF is impaired in dialysis patients and may be associated with poor gastrointestinal tolerance. The consensus report on therapeutic drug monitoring of MPA in solid organ transplantation recommend a therapeutic target window of area under the plasma concentration–time curve from 0 to 12 hours (MPA-AUC_0-12_) of 30–60 mg h/L in calcineurin inhibitor-treated patients ^(56)^. However, the target MPA-AUC_0-12_ in RDs is controversial. Daleboudt et al. ^(57)^ showed that a target MPA-AUC_0-12_ of 60–90 mg h/L was associated with optimized MPA exposure and excellent renal outcomes at 12 months of follow-up in a small sample of patients with LN after treatment with low-dose intravenous pulse CYC (IVCY). Hao Bao et al. ^(58)^ maintained an MPA-AUC_0-12_ of 20–45 mg h/L in patients with class IV + V LN treated with MMF, tacrolimus (TAC), and steroid (multi-target therapy). The calculation of AUC requires more than eight blood samples. A limited sampling strategy (LSS) using the C2h-C4h-C9h time points is more feasible and easy to implement in patients treated with MMF and TAC ^(59)^. Few studies on LSS in patients treated with MMF but without calcineurin inhibitors have been reported. LSS using the C0h-C1/2h-C1h-C2h time points showed a very good correlation with MPA-AUC_0-12_ in liver transplantation ^(60)^.

### CYC

CYC has been used for many years for various RDs. A guideline for the management of adults with AAV recommends a standard IVCY dose of 15 mg/kg, which should be reduced for patients with advanced age and decreased renal function based on the protocol of the CYCLOPS study ^(61)^ (Table 2). On the other hand, the National Institutes of Health and the Euro-Lupus Nephritis protocols for patients with LN do not require IVCY dose adjustment. The CYCLOPS study excluded patients with serum creatinine of > 5.7 mg/dL (500 μmol/L), and the mean serum creatinine level was 2.55 mg/dL (225 μmol/lL). Although the study adopted the National Institutes of Health protocol for the first time, it did not have a serum creatinine cut-off for exclusion and the mean level was 1.20 mg/dL (106 μmol/L) ^(62)^. In contrast, the Euro-Lupus Nephritis Trial excluded ESRD patients with LN, and the mean serum creatinine level was 1.15 mg/dL (101 μmol/L). ESRD patients have reduced systemic clearance of CYC with a prolonged elimination half-life, and even in patients with LN, IVCY dose adjustment may be required for patients with high serum creatinine levels. In addition, synchronization of IVCY and hemodialysis should be considered. In hemodialysis patients, on average, 22% of the administered CYC dose was eliminated by a three-hour hemodialysis session starting 7 hours after CYC administration ^(63)^. Thus, IVCY should be administered after dialysis.

**Table 2.** Recommended Dosages of Immunosuppressive Agent in Patients with ESRD.

Agent	Recommended dosage
Hydroxychloroquine	Both dosage and screening frequency need to be adjusted
Mycophenolate mofetil	TDM is recommended. The target MPA-AUC (0-12 h) concentration in rheumatic disease is controversial
Cyclophosphamide	Dose adjustment may be required for patients with high serum Cr and/or old age. Intravenous CYC infusions 15 mg/kg/pulse in patients <60 years with low Cr (150–300 μmol/L or 1.7–3.4 mg/dL). 12.5 mg/kg/pulse in patients with 60–70 years and low Cr. 10.0 mg/kg/pulse in patients >70 years with low Cr. 12.5 mg/kg/pulse in patients <60 years with high Cr (300–500 μmol/L or 1.7–3.4 mg/dL). 10.0 mg/kg/pulse in patients with 60–70 years and high Cr. 7.5 mg/kg/pulse in patients >70 years with low Cr.
Cyclosporine	TDM is recommended. Trough concentration should not exceed 200 ng/mL.
Tacrolimus	TDM is recommended. Trough concentration should not exceed 20 ng/mL.
Azathioprine	CCr > 50 mL/minute, no dose adjustment recommended; CCr 10–50 mL/minute, 75% of normal dose; CCr < 10 mL/minute, 50% of normal dose Patients on hemodialysis (–45% removed in 8 hours by dialysis): 50% of normal dose for children; for adults, 50% of normal dose and supplement of 0.25 mg/kg after hemodialysis on dialysis days
Methotrexate	Contraindicated
Leflunomide	Dose adjustment is not required
Sulfasalazine	Dose adjustment is not required
Iguratimod	Careful administration is required
Bucillamine	Contraindicated
Rituximab	Dose adjustment is not required
Belimumab	Dose adjustment is not required

TDM = therapeutic drug monitoring, MPA-AUC = Mycophenolic acid-area under the curve, ESRD = end-stage renal disease, Cr = creatinine, CCr = creatinine clearance

### CSA and TAC

CSA and TAC have also been used for various RDs. These agents undergo minimal renal elimination and their mean clearance in patients with ESRD was similar to that in patients with normal renal function. However, careful monitoring of eGFR is recommended to predict nephrotoxicity. Manifestation of calcineurin inhibitor nephrotoxicity in transplant patients is subdivided into the following two types: acute azotaemia, which is usually reversible after reducing the dose, or chronic progressive renal disease, which is usually irreversible ^(64)^. Recent recommendations for the use of CSA in RA state that the starting dose should be 2.5–3.5 mg/kg/day and that the maximum dose should not exceed 5 mg/kg/day. Dose reduction is necessary whenever serum creatinine increases by more than 30% ^(65)^. Dose adjustment should be performed when a trough concentration is higher than 200 ng/mL to prevent nephrotoxicity ^(66)^. Bottiger et al. ^(67)^ reported that the incidence of renal impairment and infection increases when the TAC trough level in whole blood is ≥10 ng/mL in transplant recipients. Appropriate therapeutic drug monitoring is useful for optimizing TAC doses when the trough concentration is higher than 10 ng/ml ^(67)^. Dose adjustment is especially important in amyopathic dermatomyositis patients with interstitial pneumonia, who should be initially treated at a 10–20 ng/mL TAC trough level. Although multiple agents to minimize nephrotoxic effects have been evaluated, none was clearly effective.

### AZA

Although dose reductions are recommended, specific guidelines are not available in the FDA-approved labeling. Published recommendations are shown in Table 2 ^(68)^.

### MTX

MTX is contraindicated in ESRD patients because it causes severe bone marrow suppression and neutropenia ^(69)^.

### Other disease-modifying antirheumatic drugs

Leflunomide can be used in patients with ESRD without significant dose reduction due to the stable concentration of its active metabolite, teriflunomide, which is only partially removed by dialysis ^(70)^. Sulfasalazine has also been reported to be safe in RA patients with ESRD after titration to full therapeutic doses ^(71)^. The safety of iguratimod in patients with ESRD is unclear; however, Takasugi et al. ^(72)^ reported that it can be safely used in patients with eGFR < 60 mL/min. According to the Japanese package insert, careful administration is required in patients with renal impairment. Bucillamine is contraindicated in patients with renal diseases due to the possibility of drug-induced nephrotic syndrome, according to the Japanese package insert.

### Biological agents

Rituximab in combination with corticosteroids is recommended in ANCA-associated vasculitis. In LN patients who fail to respond to either MMF or CYC, shifting to another regimen or rituximab should be considered ^(54)^. Rituximab is not eliminated by hemodialysis and can be used in these patients without dose adjustment. Belimumab, a new therapeutic agent for SLE, is a fully humanized IgG1-λ monoclonal antibody that binds to soluble B lymphocyte stimulator and inhibits its binding to its receptors, and thus its activity ^(73)^. A pooled analysis of four studies of intravenous belimumab in 1,603 patients with SLE found only 14 subjects with severe renal impairment of creatinine clearance of ≥15 to <30 mL/min. Although increased creatinine clearance and proteinuria (>2 g/day) are associated with increased belimumab clearance, these effects were not clinically significant. Therefore, dose adjustment in patients with renal impairment is not recommended ^(74)^.

## The Role of Rheumatologists in the Management of ESRD Patients

A team approach involving rheumatologists and nephrologists is expected to produce a complementary effect and achieve better outcomes. A retrospective study showed that frequent follow-up visits by rheumatologists, at least twice a year, improved longevity in patients with SLE on renal replacement therapy and meant that the patients were more likely to receive effective immunosuppressive therapy ^(75)^. Rheumatologists can support nephrologists during unfamiliar clinical situations and vice versa. First, rheumatologists should investigate extra-renal manifestations on physical examination suggestive of RD relapse, such as cutaneous and mucosal lesions, arthralgia arthritis, myalgia myositis, lymphadenopathy, and motor and sensory disturbances. Second, rheumatologists should assess activity of RDs and the need for adjusting treatment. Physical examination on a regular basis is pertinent because serologic parameters may not serve as accurate markers of disease activity and may not help predict disease recurrence in patients with ESRD. Third, rheumatologists should adjust not only the doses of corticosteroids, but also those of immunosuppressive agents such as hydroxychloroquine, MMF, and CYC, if necessary.

The progress in medicine has prolonged the life expectancy of ESRD patients, with the increased survival accompanied by a risk of RD relapse and development of other comorbidities. Collaboration between rheumatologists and nephrologists is more important than ever to improve quality of life in patients with renal impairment due to RDs.

## Article Information

### Conflicts of Interest

H.Y. received consultant fees, speaker fees, research grants, scholarship donations, or donations to the research department from MSD, Ayumi, AbbVie, Eisai, Ono, Astellas, Daiichi-Sankyo, Taisyo-Toyama, Takeda, Tanabe-Mitsubishi, Chugai, Teijin Pharma, Torii, Nippon Shinyaku, Pfizer, UCB, Nippon Kayaku, YL biologics, Bayer, and Bristol-Meyers; M.H. Tokyo Women’s Medical University (TWMU), particularly the Division of Epidemiology and Pharmacoepidemiology in Rheumatic Diseases, has received unrestricted research grants from Ayumi Pharmaceutical Co., Chugai Pharmaceutical Co., Ltd., Eisai Co., Ltd., Nippon Kayaku Co., Ltd., Taisho Toyama Pharmaceutical Co., Ltd., Takeda Pharmaceutical Co., Ltd., Mitsubishi Tanabe Pharma Co., and Teijin Pharma Ltd., with which TWMU paid the salary of M.H.

### Sources of Funding

This work was supported by the research grants to M.H. from the Ministry of Health, Labour, and Welfare of Japan grant number [H26-nanchi-nanchitou (nan)-ippan-044].

### Author Contributions

Suguru Honda wrote the initial draft of the manuscript. Masayoshi Harigai assisted in the preparation of the manuscript. All other authors have contributed to data or reference acquisition and critically reviewed the manuscript. All authors approved the final version of the manuscript and agree to be accountable for all aspects of the work in ensuring that questions related to the accuracy or integrity of any part of the work are appropriately investigated and resolved.

### Approval by Institutional Review Board (IRB)

Not required

### Disclaimer

Masayoshi Harigai is one of the Editors of JMA Journal and on the journal's Editorial Staff. He was not involved in the editorial evaluation or decision to accept this article for publication at all.
